# Quantitative evaluation of genotoxicity data for risk assessment and regulatory decision-making: Time for a paradigm shift

**DOI:** 10.1016/j.envint.2026.110070

**Published:** 2026-01-16

**Authors:** Benjamin Sachse, Sebastian Schmeisser, Jan van Benthem, Raffaella Corvi, Eugenia Dogliotti, Norman Ertych, Roland Frötschl, Ulrike Gündel, Kristin Herrmann, George Johnson, Carsten Kneuer, Jeannette König, Hans-Jörg Martus, Stefan Pfuhler, Stephanie Smith-Roe, Helga Stopper, Paul White, Tanja Schwerdtle, Andreas Hensel, Tewes Tralau

**Affiliations:** a German Federal Institute for Risk Assessment, Max-Dohrn-Straße 8–10, 10589 Berlin, Germany; b The National Institute for Public Health and the Environment, Antonie van Leeuwenhoeklaan 9, 3721 MA Bilthoven, the Netherlands; c European Commission, Joint Research Centre, Via E. Fermi, 2749, 21027 Ispra, Italy; d Department of Environment and Health, Istituto Superiore di Sanità, 299, Viale Regina Elena, 00161 Rome, Italy; e Federal Institute for Drugs and Medical Devices, Kurt-Georg-Kiesinger-Allee 3, 53175 Bonn, Germany; f Swansea University Medical School, Swansea University, Swansea, SA35DE Wales, UK; g Novartis Biomedical Research, Lichtstrasse 35, 4056 Basel, Switzerland; h Procter & Gamble, Global Product Stewardship – Human Safety, Cincinnati, OH, USA; i Division of Translational Toxicology, National Institute of Environmental Health Sciences, Research Triangle Park, NC, USA; j Institute of Pharmacology and Toxicology, University of Wuerzburg, Versbacher Str. 9, 97078 Wuerzburg, Germany; k Environmental Health Science and Research Bureau, Health Canada, 251 Sir Frederick Banting Driveway, Ottawa, Ontario K1A0K9, Canada; l Max Rubner-Institut, Federal Research Institute of Nutrition and Food, Haid-und-Neu-Straße 9, 76131 Karlsruhe, Germany

**Keywords:** Genotoxicity, Mutagenicity, Quantitative approach, Regulatory toxicology, Risk assessment, Symposium

## Abstract

Genotoxicity plays an important role in chemical safety assessment, as genetic alterations can lead to severe and irreversible health consequences. To date, the assessment of genotoxicity has mostly been limited to hazard identification, followed by rigorous risk mitigation measures if a substance is found to be mutagenic, regardless of potency, the underlying mechanism, and cellular biology. While this regulatory hazard-based approach is straightforward, it is unsatisfactory when exposure to genotoxic substances cannot be completely avoided and/or regulatory measures lead to misperceptions of risk and undesirable socioeconomic side effects. The latter becomes particularly obvious in light of natural genotoxicants, *e.g.* occurring in plant-based food, and for substances that are difficult to replace but come with a high socioeconomic value but little potency and exposure. Hence, there is an increasing demand for a paradigm shift towards a quantitative interpretation of genotoxicity data in regulatory risk assessment. However, moving away from the traditional hazard-based assessment and doing so safely requires a collective effort of all relevant stakeholders. To this end, the German Federal Institute for Risk Assessment (BfR) organised an international symposium, at which experts from regulatory authorities, academia and industry discussed the opportunities and challenges involved. Here, we present key issues to be considered for a successful implementation of quantitative approaches. In situations where exposure to genotoxic substances cannot be completely avoided, *e.g.* occurrence of contaminants, quantitative approaches offer the opportunity to better characterise the associated risks and thus enable risk managers to make more informed decisions.

## 1. Introduction

Maintaining DNA integrity is essential for the health of individuals and populations, so a comprehensive and finely regulated system of checks and comprehensive DNA repair systems exist in most organisms to prevent permanent genetic alterations. Although modifications of the DNA do not necessarily equate to detrimental genetic alterations, they can result in adverse outcomes if critical genes are affected, and damage is not adequately repaired. In somatic cells, this comprises most prominently cancer but also other diseases, for example of the cardiovascular system (Heimlich & Bick 2022). Even more critical are mutations in germ cells, where theoretically a single mutation can lead to heritable damage to the offspring or future generations (OECD 2017; WHO 2020b). Thus, robust assessment of genotoxic potential remains central to regulatory frameworks worldwide, supporting efforts to protect human health, environmental health, and biological stability. Indeed, genotoxicity assessment is a cornerstone of chemical safety assessment, providing critical insights into the potential for chemicals to cause genetic alterations that could cause heritable genetic damage and other adverse health outcomes (Heflich et al. 2020; Parsons et al. 2024).

The term genotoxicity refers to any damage to the genetic material, including defects that can be repaired as well as irreversible changes. The more specific term mutation “refers to permanent changes in the structure or amount of the genetic material of an organism that can lead to heritable changes in its function; these changes include gene mutations as well as structural and numerical chromosomal alterations” (WHO 2020b).

Genotoxic damage can be caused by exogenous agents, *e.g.* chemicals. In addition, genotoxic damage may also occur spontaneously or as a result of endogenous stressors, for instance, during DNA replication or as a result of metabolic processes that generate reactive oxygen species (ROS) or endogenous metabolites, such as formaldehyde or acetaldehyde. Genotoxicants are often subdivided into DNA-reactive substances that interact directly with the genetic material, *e.g.* by forming covalent DNA adducts or crosslinks, and non-DNA-reactive substances causing damage to the DNA through indirect mechanisms, such as the generation of ROS or by disruption of mechanisms that maintain genomic stability or disruption of the spindle apparatus during replication. Networks of DNA damage response mechanisms generally enable effective repair to maintain genomic stability. The effectiveness of these repair mechanisms strongly depends on the extent and molecular cause of damage. Notably, given the plethora of natural DNA-modifying agents, DNA-repair is necessarily efficient and efficacious. Yet, in replicating cells, genotoxic damage can – unless adequately repaired – be converted into irreversible mutations or lead to cell death (Pratt & Barron 2003; Jackson & Bartek 2009; Kunkel & Erie 2015; Chatterjee & Walker 2017; WHO 2020b).

Genotoxicity assessment has traditionally followed a hazard-based approach rooted in the so-called one-hit or single-hit hypothesis. This hypothesis states that even a single reactive molecule, capable of damaging DNA, can induce a genetic defect. A defect that is fixed (made permanent), for example in the form of a coding mutation in a cancer-related gene, can be transmitted to the daughter cell and ultimately initiate cancer (Golden et al. 2019) or other adversities. For DNA-reactive compounds, this paradigm not only assumes a strict linear dose–response relationship in the low-dose range, and completely disregards the possibility of a biologically meaningful or practical threshold for genotoxic responses, but it also operates under the assumption of (continuous) additivity. In consequence, if a substance has been shown to be genotoxic *in vivo*, it is assumed that any exposure to the substance is associated with DNA damage, leading to an increased risk of mutations and, in turn, downstream adverse human health effects (COM 2010). Historically, this assumption originated from radiation biology and was later transferred to genetic toxicology (Golden et al. 2019). In its strict interpretation there is by definition no safe level of exposure for DNA-reactive genotoxic substances. By contrast, a threshold is generally assumed for indirectly acting genotoxicants, and a safe exposure level can be determined in principle. However, this has rarely been done in regulatory practice in the past, even for such agents. This is because extensive research is required to determine the exact mechanism(s) behind a genotoxic response and the actual threshold. Overall, in most cases, quantitative assessments are only conducted on the basis of animal data for apical endpoints, *e.g.* from carcinogenicity studies, if such data is available.

Risk management consequences for genotoxic substances differ across jurisdictions and sectors but often converge on a conservative approach, generally aimed at minimising exposure. In European contexts, the ALARA (as low as reasonably achievable) principle has been widely referenced, particularly for food-related exposures. This concept is used because it reflects the assumption of a linear, non-threshold dose–response relationship at low doses and accounts for the potential severity of health effects associated with genotoxicants, as well as the inherent uncertainties associated with their assessment, *e.g.* effects potentially resulting from combined exposure to multiple genotoxicants (EFSA 2005; Menz et al. 2023). In contrast, the United States and Canada often apply a *de minimis* risk framework in food and environmental contexts, where a lifetime cancer risk, for example, below 10^–6^ (one in a million) is typically considered negligible and may not require additional regulatory action (EPA 1996; Health Canada 2024). Overall, while terminology and numerical benchmarks may differ, global regulatory frameworks generally favour conservative, precautionary approaches to genotoxicity – often assuming no safe threshold.

Although often used for practical reasons, the single-hit hypothesis lacks consideration of biological relevance, for example, because it ignores the continual formation of endogenous DNA damage and the effective mechanisms in place to repair it (Calabrese 2019). In fact, the linear no-threshold paradigm is challenged by a growing body of evidence suggesting that indirectly as well as directly acting genotoxicants exhibit non-linear dose–response relationships (Müller & Gocke 2009; ECHA 2018; Fukushima et al. 2018; Hartwig et al. 2020; EFSA 2021b; Johnson et al. 2021). Although this has partly been acknowledged in some regulatory guidance documents (ECHA 2017; WHO 2020b; EFSA 2021b; EMA 2023), there is currently no officially adopted methodology for assessing the risk of (low) exposures to DNA-reactive genotoxicants on the basis of genotoxicity data. The only regulatory concepts concern aneugens (EFSA 2021b) and the use of a Permissible Daily Exposure (PDE) approach for indirect (non-DNA-reactive) genotoxicants as outlined in ICH M7 for pharmaceuticals (EMA 2023).

Nevertheless, the regulatory utility of the linear no-threshold paradigm has been challenged. Together with the fact that complete avoidance of exposure to genotoxic substance is not possible, and that strictly hazard-based approaches can lead to undesired socioeconomic side effects, an increasing demand for a paradigm shift towards a more quantitative assessment paradigm for genotoxic substances appears necessary. In this context, data-supported derivation of genotoxicity thresholds can support risk managers dealing with the risk of genotoxicity in the wider context of health protection.

In 2022, the German Federal Institute for Risk Assessment (BfR) weighed in on the debate by publishing a comprehensive review on opportunities, challenges and perspectives for quantitative evaluations of genotoxicity dose–response data (Menz et al. 2023). Subsequently, in February 2024, BfR continued the discussion by organising and hosting the “International Symposium: Risk assessment of genotoxic compounds: Challenges and Future Perspectives” in Berlin. The focus was to bring together experts from different disciplines and regulatory paradigms to discuss options for quantitative approaches in future genotoxicity assessment. The symposium took place over 3 days, beginning with introductory lectures that provided an overview of the current assessment strategies and options for quantitative procedures. This was followed by presentations from representatives of several international authorities (European Commission, European Food Safety Authority, European Chemicals Agency, U.S. Food and Drug Administration, U.S. National Institute of Environmental Health Sciences, Health Canada, German Federal Institute for Drugs and Medical Devices) about their perspectives on quantitative genotoxicity assessment. The topic was then intensively discussed in four workshops addressing 1) risk assessment of genotoxic compounds in the presence of data from carcinogenicity studies, 2) quantitative assessment of genotoxic compounds in the absence of data from carcinogenicity studies, 3) new approaches for the characterisation of genotoxic effects based on *in vitro* and *in silico* data, and 4) new approaches for the quantitative characterisation of genotoxic effects based on animal and human data. Conclusions were drawn in a panel discussion with high-level representatives from authorities, academia, and industry.

A perspective on quantitative genotoxicity assessment is presented here, reflecting the discussions held at the BfR symposium and the ongoing debate within the regulatory community. It illustrates the current status quo, explains the reasons for the need to evaluate and adapt the traditional paradigm, and provides practical steps needed to improve the acceptance of quantitative assessments in genotoxicity to incorporate them in chemical safety assessment. While this perspective outlines technical aspects for expert discussions, it is also intended to stimulate a broader societal discussion about the question of acceptable levels of risk and uncertainty.

### 1.1. Status quo

#### 1.1.1. Hazard identification

Genotoxicity assessment is an important part of chemical safety assessment. In almost all regulatory contexts, it is one of the first steps in the assessment.

Genotoxicity testing is generally carried out in a tiered approach. In a first step, different genotoxic events, *i.e.* gene mutations, structural chromosomal aberrations (clastogenicity), and numerical chromosomal aberrations (aneugenicity), are typically evaluated by an *in vitro* test battery, as no single test can currently detect these endpoints simultaneously. This tiered approach usually includes the bacterial reverse mutation assay (OECD TG 471) to detect gene mutations, and the *in vitro* micronucleus assay (OECD TG 487) to cover clastogenic and aneugenic effects. An assay to test for gene mutations in mammalian cells may also be performed, *e.g.* the *in vitro* mammalian cell gene mutation tests using the *HPRT* or *XPRT* genes (OECD TG 476) or the thymidine kinase (*TK*) gene (OECD TG 490). The *in vitro* mammalian chromosome aberration test (OECD TG 473) may also be used to detect clastogenicity. However, depending on the regulatory area, recommendations as to which tests are preferred are slightly different (FDA 2000; EFSA 2011; EMA 2012; ECHA 2017; COM 2021; Health Canada 2022).

If the results obtained with the entire test battery are negative, no further testing is required in many regulatory areas, and it is assumed that the test substance does not have a relevant genotoxic potential. However, there are still some sectors, *e.g.* pharmaceuticals, in which it is mandatory to carry out *in vivo* testing, even if the results of *in vitro* tests are negative. Exceptions may also be made if the available data indicate that *in vitro* testing is inadequate. In contrast, positive results generally require follow-up testing in appropriate *in vivo* assays. The choice of the appropriate follow-up test depends on the type of genotoxic event identified *in vitro*. Relevant test systems include the transgenic rodent somatic and germ cell gene mutation assays (OECD TG 488), the *Pig-a* gene mutation assay (OECD TG 470), the *in vivo* mammalian alkaline comet assay (OECD TG 489), the mammalian erythrocyte micronucleus test (OECD TG 474), the mammalian bone marrow chromosome aberration test (OECD TG 475) or a combination of these assays (EFSA 2011; EMA 2012; ECHA 2017; COM 2021).

New approach methodologies (NAM) and non-guideline tests, which are not currently part of the standard test battery, can provide additional information to better understand the mode of action (MoA) underlying the observed genotoxic effects and may be considered as supporting data in the safety assessment on a case-by-case basis (EMA 2012; ECHA 2017; WHO 2020b). Similarly, when a test substance that is not available in sufficient quantities for testing or a prioritisation decision has to be made from a large group of candidate substances, *in silico* predictions for parent compounds and their known or predicted metabolites based on computational models are increasingly applied.

A final decision on genotoxic properties is generally made in a weight-of-evidence (WoE) approach by taking into account all available information and considering provisions from specific guidance documents (*e.g.* EFSA 2023). Traditionally, genotoxicity data have been interpreted mainly in a qualitative manner, generally resulting in a binary categorisation of chemical entities as being genotoxic or non-genotoxic. In some regulatory areas (Table 1), such as chemical safety assessment under the Regulation on the Registration, Evaluation, Authorisation and Restriction of Chemicals (REACH, EC No 1907/2006) or the Biocidal Products Regulation (EU No 528/2012), genotoxicity data are generated and assessed to appropriately classify and label a substance. Corresponding classification criteria for the hazard class “germ cell mutagenicity” (Muta 1A, 1B, or 2) are laid down in the overarching EU Regulation on Classification, Labelling, and Packaging (CLP, EC No 1272/2008) of Substances and Mixtures (EFSA 2011; ECHA 2017; WHO 2020b).

#### 1.1.2. Risk characterisation

Health risks resulting from genotoxic agents are usually assessed in a qualitative way, and positive results from *in vivo* genotoxicity studies trigger rigorous risk management measures, irrespective of potency. Quantitative assessments are only accepted on the basis of animal data for apical endpoints, *i.e.* from carcinogenicity studies. For substances that are both genotoxic and carcinogenic and for which carcinogenicity data are available, either the margin of exposure (MoE) approach or the derived minimal effect level (DMEL) approach is commonly used for this purpose in Europe (EFSA 2005; ECHA 2012).

The MoE approach uses a point of departure (PoD, synonymously: toxicological reference point) from animal studies and relates it to the estimated human exposure. The higher the MoE, the lower the health concern. An MoE of 10,000 or more is often considered to be of low concern from a public health point of view, if it is based on the BMDL_10_ from a carcinogenicity study in animals (*i.e.* a PoD that indicates the lower bound of the estimated dose corresponding to a response of a 10 % excess tumour incidence). However, even such exposure levels are generally not considered safe. Instead, the MoE provides an estimate of the level of concern associated with a particular exposure. It also serves as a tool for communication to risk managers and is intended to enable them to prioritise risk management measures. The MoE approach is widely used throughout different regulatory areas in Europe and other countries (*e.g.* Canada) (EFSA 2005; Health Canada 2021). A modified MoE approach is currently applied for farm animals and pets with regard to exposure to botanically defined flavouring substances containing genotoxic and/or carcinogenic substances for authorisation as feed additives (EFSA 2021a).

In comparison, the DMEL (derived minimum effect level) approach for non-threshold (genotoxic) carcinogens aims to derive “an exposure level corresponding to a low, possibly theoretical, risk”, albeit based on the same data. The associated dose can be derived either by the “large assessment factor approach”, which takes into account certain assessment factors, or by the linearised approach, which uses linear extrapolation (*e.g.* based on the T25 value, *i.e.* the dose that leads to an additional tumour incidence of 25 % above the control). For the latter approach, an additional cancer risk of 10^−5^ or 10^−6^ is often considered a tolerable risk, for workers and the general population, respectively (ECHA 2012). When using the large assessment factor approach, an overall assessment factor of 10,000 is typically used to derive the DMEL for the general population (ECHA 2012). It should be noted, however, that linear extrapolation from cancer studies to the human situation needs to be critically reviewed as it may not reflect the actual risk (EFSA 2005; ECHA 2012). The DMEL approach is commonly applied in chemical safety assessments under REACH (Table 1), but it is uncommon in many other regulatory frameworks, such as food safety in Europe.

The U.S. Food and Drug Administration (FDA) applies a risk-based approach to genotoxic impurities in food additives, distinguishing between additives and their constituents. While the Delaney Clause mandates a zero-risk standard for carcinogenic food and colour additives, FDA employs a *de minimis* cancer risk standard for constituents. This allows for cancer risk assessments under the general safety clause without triggering the Delaney Clause. FDA follows the U.S. National Research Council guidelines and Office of Science and Technology Policy principles, using a weight-of-evidence approach. For potential carcinogens, FDA conducts quantitative risk assessments, typically using linear-at-low-dose extrapolation to derive a unit cancer risk (UCR). The agency considers a lifetime cancer risk (LCR) of less than 10^−6^ as *de minimis*. For food contact substances, an LCR below 10^−6^ for cumulative exposure or 10^−8^ for incremental exposure is deemed negligible, supporting the safety standard of reasonable certainty of no harm (Zang & Kabadi 2023).

In cases where only genotoxicity data but no carcinogenicity studies are available, the threshold of toxicological concern (TTC) approach is currently the only established option to provide risk managers with some information on the need for risk mitigation measures. This approach is based on carcinogenicity data from other substances. For genotoxic compounds, a TTC value of 0.0025 µg/kg body weight (bw) has been derived by Kroes *et al.* from the “Carcinogenic Potency Database” (Kroes et al. 2007). If the exposure is below the TTC, the risk of adverse effects is considered low, and no further measures may be needed. In contrast, higher exposure levels require further testing and a more comprehensive assessment. Whereas the TTC approach is widely used for contaminants and impurities, it is generally not intended for intentionally used substances like pharmaceuticals or pesticide active substances. Furthermore, it should not be used for high-potency genotoxicants, such as aflatoxins, azoxy, and *N*-nitroso compounds (FSANZ 2013; EFSA 2019a; EMA 2023).

In cases where no carcinogenicity data are available, quantitative assessments based on genotoxicity data are mostly not accepted. However, they can be considered in cases where genotoxicity is caused by indirect mechanisms, such as molecular interactions with cellular processes (*e.g.* microtubule assembly) whose underlying molecular mechanisms are sufficiently understood. As outlined in more detail in section 2 of this manuscript, this has already been implemented in a number of assessments, yet it remains an exception in regulatory practice. For aneugens, if a threshold-based MoA can be clearly demonstrated, adequate dose–response information is available, and it is proven that no other non-threshold genotoxic MoA is involved, a quantitative risk characterisation is already acknowledged in regulatory guidance documents (ECHA 2017; EFSA 2021b; EMA 2023). As the available data are often not sufficient to clearly demonstrate that genotoxicity is solely based on indirect mechanisms, genotoxicants are by default assumed to have no threshold in most regulatory areas. Quantitative assessments for other indirect genotoxic mechanisms, such as interference with DNA synthesis, DNA repair processes, or cellular defence systems are not routinely performed.

#### 1.1.3. Regulatory aspects

Table 1 summarises some aspects described above related to hazard and risk assessment procedures and some regulatory consequences for genotoxic substances for different selected regulatory areas within the European Union. As shown in the table, most regulations for the hazard assessment of genotoxic substances are based on the classification provisions of the CLP Regulation. Consequently, if substances fulfil the classification criteria for the hazard class “germ cell mutagenicity” category Muta 1A/B, then they are not allowed on the market unless derogation criteria based on exposure considerations or socioeconomic needs are applied to justify their use. For substances classified as germ cell mutagens, usually no reference values for the safe use can be established and use restrictions may be triggered. However, if a threshold-based MoA can be clearly demonstrated for genotoxic substances (such as aneugens), the derivation of a DNEL (Derived No Effect Level) (ECHA 2012) is possible and has already been implemented in some regulations. For genotoxic substances without a threshold-based MoA, a DMEL (Derived Minimal Effect Level) can be calculated based on carcinogenicity data – if available – to support regulatory decisions at the risk management level.

Of note, there are regulatory areas such as mixture classification under the CLP Regulation where, for the sake of manageability, the application of pragmatic thresholds is generally accepted for genotoxic substances. The CLP regulation classifies mutagens into those either “known” or “to be regarded as if they” induce heritable mutations in germ cells of humans (Muta 1A or Muta 1B) and substances that cause concern owing to the possibility that they may induce heritable mutations in the germ cells of humans (Muta 2). Unless there are data on a mixture itself demonstrating mutagenic effects, mixtures containing a mutagen classified as Muta 1A/B or Muta 2 below a generic concentration limit (GCL) of 0.1 % or 1 %, respectively, do not need to be classified or labelled (Table 1, CLP Regulation, Annex 1, Section 3.5.3) (EC 2008). The same applies to multi-constituent substances, *i.e.* substances with mutagenic impurities or additives. Notably, the above-mentioned GCLs of 0.1 or 1 % are applied to all mutagens irrespective of their potency. The current CLP Guidance considers it impossible to set substance specific concentration limits (SCLs) for mutagens, referring in particular to the inability of current test methodology to provide the required dose–response information.

### 1.2. Need to assess and adapt the traditional paradigm

Qualitative assessment strategies for genotoxic compounds have been used for several decades. However, the procedure suffers from several drawbacks and the resulting binary categorisation as genotoxic or non-genotoxic triggers far-reaching consequences without distinguishing the potency of genotoxicants and their potential thresholds. Current quantitative approaches that involve genotoxicants are generally based on apical data from *in vivo* carcinogenicity studies in laboratory animals. These studies may become less relevant with the development towards next-generation risk assessment (NGRA) practices that are mainly informed by non-animal data on early and intermediate effects. With the development of a roadmap for phasing out animal testing in chemical safety assessments, the European Commission is currently outlining milestones and concrete actions for a transition to animal-free chemicals legislation. Similar activities are ongoing in other countries (FDA 2025; Health Canada 2025). Moreover, most regulatory bodies are currently implementing the 3Rs (reduce, refine, or replace) into safety assessments to minimise the use of animals unless proven scientifically necessary.

The traditional approach dates back to a time when genotoxicity testing was in its infancy and only a few genotoxicants were known (Calabrese 2019; Clewell et al. 2019). At that time, the prevailing view may have been that banning or avoidance of such substances is relatively straightforward in order to protect human health from potentially serious consequences caused by genotoxicants. However, this assumption is now challenged by improved analytical methods revealing the ubiquity of these substances in our environment – in air, water, food, and consumer products. These substances include synthetically produced chemicals but also numerous naturally occurring substances and contaminants (Cartus & Schrenk 2017; White et al. 2020; Bartoszek & Holota 2023). The latter ones are often impossible to avoid, and risk management measures taken to reduce exposure to such compounds are often associated with significant undesirable side effects, *e.g.* negative socioeconomic consequences (Habs et al. 2017; Williams 2019).

Many foods contain natural genotoxic constituents or contaminants, sometimes in low concentrations, some of them with moderate activity, others more potent (Jägerstad & Skog 2005; O’Brien et al. 2006; van den Berg et al. 2011; Bartoszek & Holota 2023). Most current assessment strategies do not account for these potency differences when carcinogenicity data are unavailable. This situation creates a paradox: foods consumed for centuries might face market restrictions due to the detection of genotoxic substances, despite no clear evidence of health risks. From a toxicological and consumer protection standpoint, this approach is debatable, as completely avoiding exposure to natural genotoxicants (*e.g.* alkenyl benzenes or pyrrolizidine alkaloids, which are secondary plant metabolites present in foods; acrylamide; furan; arsenic; polyaromatic hydrocarbons; nitrosamines; and mycotoxins such as aflatoxin B1) would require abstaining from most foods, disregarding the complex balance of nutritional risks and benefits. In this context, a quantitative assessment taking into account all available information seems desirable, not only to provide useful information for risk managers but also to handle risks related to genotoxic and mutagenic substances more appropriately.

As another example, many disinfection protocols rely on the use of chemically reactive aldehydes (*e.g.* formaldehyde and its releasers, glutaraldehyde), oxidants (*e.g.* chlorine, peroxides, peracetic acid) or alkylating agents (ethylene oxide, methyl bromide) with an inherent potential for genotoxicity. A complete ban of these substances would increase the obvious health risk from the respective pathogens. A restricted inventory of biocides with less diversity regarding MoA and chemical structures would reduce the available options to tackle microbial resistances. Given the often severe health consequences associated with the organisms targeted by genotoxic biocides, quantitative assessments may allow the determination of safe use levels or, when a zero-risk exposure cannot be defined – support a risk–benefit analysis to allow for informed public health management decisions.

Furthermore, as described above, mixtures containing a mutagen classified as Muta 1A/B or Muta 2 below a GCL of 0.1 % or 1 %, respectively do not need to be classified or labelled. This common practice may indeed create a safety concern, as *e.g.* for potent Muta 2 classified somatic cell mutagens a GCL of 1 % for mixture classification may not be protective. Quantitative assessments could lead to more specific potency-based limits, for example for mixtures that are intended for consumer usage. Currently, however, no accepted guidance is available for the setting of SCLs for mutagenicity (Guidance on the application of the CLP criteria, Part 3: Health hazards) (EC 2008).

As abovementioned, genotoxicants are generally assessed qualitatively unless carcinogenicity data are available that can be used within, *e.g.* a MoE or DMEL approach. This focus on carcinogenicity data for a quantitative assessment of genotoxic substances is being critically questioned. The probability of apical effects, such as cancer caused by genotoxic noxae, increases with increasing dose. Therefore, at doses that are reflective of human exposures, tumour incidences will generally be low. To be able to detect such effects in a standard rodent carcinogenicity study, commonly using 50 animals per dose group and sex, high doses up to the maximum tolerated dose are required that usually lie orders of magnitude above the expected human exposure levels. However, at high-dose levels, unspecific effects like cytotoxicity and regenerative tissue growth may co-occur and promote the carcinogenic effects, which may lead to an overestimation of the carcinogenic risk and, therefore, limits the (quantitative) human relevance of these studies. In addition, there is a growing interest in avoiding long-term carcinogenicity studies, which are generally associated with an increased level of animal suffering, for animal welfare reasons (Cohen 2017; Corvi et al. 2017; Goodman 2018; Felter et al. 2020). Even more important, carcinogenicity studies are often not available and may only be requested in certain regulatory areas. Moreover, it is well known that genotoxicity can lead to a wide range of adverse health effects in addition to cancer. As a consequence, it has been convincingly argued that genotoxicity should be considered as a *bona fide* endpoint (Heflich et al. 2020; Parsons et al. 2024). The quantitative interpretation of genotoxicity data will enable characterisation of the risk of occurrence of the genotoxic event itself (initiating event). According to the adverse outcome pathway (AOP) concept, adverse downstream health outcomes are also not expected at levels below which genotoxic effects do not occur (Moore et al. 2018).

Moving away from the binary approach towards a quantitative risk-based assessment could enable more informed decision-making in some regulatory contexts. Quantification should be provided both for the dose–response relationship as well as the (un)certainty / confidence associated with the classification and the corresponding dose–response function. This would also be an opportunity to avoid improper risk perception, disproportional risk mitigation measures and unnecessary socioeconomic impact. Nevertheless, care should be taken along the way towards a more quantitative system as there are a number of unresolved questions and uncertainties, which were discussed in the workshop and are reported herein, in need of thorough scientific evaluation before the transition can be achieved. It is imperative that human health protection must remain paramount throughout the transition process.

## 2. Workshop discussions towards a quantitative interpretation of genotoxicity data

A paradigm shift towards a quantitative interpretation of genotoxicity data seems advisable, at least for some regulatory bodies and some sectors (Schuler et al. 2011; MacGregor et al. 2015a; MacGregor et al. 2015b; White & Johnson 2016; Dearfield et al. 2017; White et al. 2020). The general idea behind the concept of quantitative genotoxicity assessment is to analyse dose–response data from genotoxicity studies, derive PoDs, and to integrate this information into the overall risk assessment, next to toxicological data from other endpoints. Taking advantage of the dose–response information may help characterise effects at low exposure levels. Attention should be paid to the biological/toxicological relevance and reliability of the data.

There is an ongoing debate over technical issues (*e.g.* the appropriate metric to derive PoDs and the suitability of data from genotoxicity studies for quantitative analysis) but also over more fundamental questions related to the biological/toxicological relevance of PoDs that are derived from genotoxicity data (White et al. 2020; Menz et al. 2023). Fig. 1 summarises the most pressing issues identified in the discourse.

In general, two conceptually different approaches for the integration of dose–response data from genotoxicity studies in the risk assessment of chemicals have emerged.

The first one considers an integration of quantitative dose–response data into a MoA-based risk assessment. The aim of such an approach is to consider all available information on the genotoxic properties of the compound under evaluation, especially with respect to the MoA and the dose–response relationship. The general assumption behind that approach is, that with enough information, either demonstrating an indirect genotoxic mechanism and/or identifying a threshold would be feasible. Based on this knowledge, it may be possible to derive limit values below which a genotoxic effect is unlikely. A decision tree method to differentiate carcinogenic substances based on their MoA has already been established in the context of setting occupational exposure limits (OELs). The Scientific Committee on Occupational Exposure Limits (SCOEL), for example, defined four groups of carcinogens: (A) carcinogens with a non-threshold MoA, (B) carcinogens that are likely to act through a non-threshold MoA, (C) carcinogens that are likely to act through a threshold MoA (*e.g.* indirect genotoxicants), (D) carcinogens with a threshold MoA (*e.g.* aneugens) (RAC/SCOEL 2017). Similarly, the German MAK Commission (The Permanent Senate Commission for the Investigation of Health Hazards of Chemical Compounds in the Work Area of the German Research Foundation) derives exposure limits (MAK values) for substances in category 4, *i.e.* for substances that are primarily non-genotoxic carcinogens or whose genotoxic effects are negligible below the derived exposure limit, as is the case, for example, with the assessment of formaldehyde (MAK 2025). Such an approach is only feasible for well-investigated chemicals, as it requires in-depth data regarding the toxic properties of the chemical, including a comprehensive quantitative characterisation of the genotoxic effects in relevant tissues and for different genotoxicity endpoints, the quantitative impact of DNA damage repair, information on the exact MoA, the connection with downstream apical effects, etc. (RAC/SCOEL 2017; Hartwig et al. 2020). There is currently no guidance regarding data requirements for such an approach. For it to work, comprehensive data from *in silico*, *in vitro* and *in vivo* studies will generally be needed – far beyond the information that are obtained in standard (geno)toxicity studies. The implementation of this approach will hence be a case-by-case decision, as done in the past for selected aneugens.

The second approach aims to use data from available genotoxicity studies for the analysis of the dose–response relationship to associate human exposure with the likelihood of an effect. These can be used for deriving a PoD associated with a (more or less) well-defined risk for genotoxic effects or downstream adversity. The PoD, in turn, can then be integrated into the risk assessment. A straightforward way of integrating such a PoD is to use it for deriving MoE values (Johnson et al. 2014; Benford 2015; Dearfield et al. 2017; COM 2018; White et al. 2020; Chepelev et al. 2023) – similarly to the procedure that is routinely conducted for genotoxic carcinogens on the basis of carcinogenicity data. Indeed, employing PoDs from genotoxicity studies in an MoE approach appears to be advantageous given that the approach constitutes an already established and accepted tool for genotoxic carcinogens. Alternatively, using genotoxicity-based PoDs to ultimately derive health-based guidance values (HBGVs) has been suggested (Johnson et al. 2014; Dearfield et al. 2017; White et al. 2020). However, the derivation of HBGVs is disputed, since a HBGV reflects “a range of exposures (either acute or chronic) that are expected to be without appreciable health risk” (WHO 2020a). Indeed, this would require a solid discussion on the uncertainty factors for genotoxicity and also a comprehensive societal discussion on which level of risk and uncertainty may be acceptable.

*In vivo* dose–response data are, at the moment, preferred to derive PoDs, as these assays inherently cover the toxicokinetics of a substance. However, a stronger consideration of *in vitro* data will become more important in the future, *e.g.* due to animal welfare and economic reasons. First experiences suggest that *in vitro* experiments may provide protective PoDs when combined with *in vitro* to *in vivo* extrapolation (IVIVE) and modelled against human exposure estimates (Kuo et al. 2022; Beal et al. 2023). Although more work is needed to establish the approach in regulatory risk assessment, it can already be used for potency ranking, especially within a well-defined chemical space (*e.g.* a group of structurally similar substances) (Wills et al. 2016). Sophisticated 3D *in vitro* models are already employed as higher tier follow-up of standard *in vitro* assays in the cosmetic sector, and the development of OECD testing guidelines for 3D skin Comet and MN is in preparation (SCCS 2023; Clewell et al. 2025). Other 3D models (*e.g.* liver, lung) may become an alternative to *in vivo* testing for regulatory purposes as well in the future (Pfuhler et al. 2020; Kopp et al. 2024).

Not only from the scientific point of view but also from a risk management perspective, an evidence-based risk assessment using extensive MoA information might be the preferred option as it may identify exposures without additional risk, provides clear reasoning for a quantitative interpretation of the data and thus increases acceptability and credibility of the assessment. As described below, the very few existing assessments that have been accepted by regulatory bodies have followed a data-driven, MoA-based approach.

### 2.1. Quantitative assessments – Current status of discussion

#### 2.1.1. Technical aspects

Although different metrics like the NOGEL (no observed genotoxic effect level), the breakpoint dose (BPD), and the BMD (benchmark dose) have been discussed as potential metrics for dose–response analyses, there is now wide consensus that the BMD approach is the preferred method for analysing dose–response data, also those from genotoxicity studies (Gollapudi et al. 2013; Johnson et al. 2014; White et al. 2020). The BMD approach makes use of all available dose–response data from a study by fitting one or multiple mathematical models to the data. Subsequently, the BMD, *i.e.* the dose that is associated with a critical effect size (CES), also known as benchmark response (BMR), can be calculated. The lower confidence limit or credibility limit of that dose (i.e the BMDL) is typically used as a PoD. This procedure is more advanced and scientifically sound than the NOGEL and BPD approach, which are not providing a definitive association between PoD and effect likelihood (MacGregor et al. 2015a; WHO 2020a; EFSA 2022). Diverging opinions were expressed on the magnitude of the CES that should be used for individual endpoints and no final conclusions have yet been reached. However, for some endpoints there are already indications regarding which CES values appear to be suitable (White et al. 2025). Further, amendments of existing genotoxicity testing guidelines (TG) to incorporate additional dose groups would be needed to improve the dose–response modelling. This can be even achieved without increasing the total number of animals, in line with the 3Rs principles of replacement, reduction and refinement (Slob 2014a, 2014b; EFSA 2022).

While some technical aspects are already advanced, others need to be further evaluated. However, it should be noted that technical aspects of dose–response analysis are not the actual factor limiting the applicability and regulatory acceptance of a quantitative interpretation of genotoxicity data.

#### 2.1.2. Biological/toxicological relevance of genotoxicity-based PoDs

Standard genotoxicity tests, both *in vitro* and *in vivo*, have been primarily designed for the identification of a genotoxic hazard. However, from a technical standpoint, as several doses are usually tested, the results can be analysed in terms of the dose–response relationship. In comparison to other toxicity tests addressing apical endpoints such as carcinogenicity, genotoxicity studies come with a number of drawbacks that limits the relevance of a quantitative interpretation of the dose–response data derived thereupon. These limitations have been comprehensively discussed by Menz *et al*. (Menz et al. 2023). For example, it can be considered critical that even *in vivo* genotoxicity studies are generally restricted to the evaluation of only one or a few selected tissues, only certain genotoxic MoA are covered in the assays, and the studies do not cover different developmental stages of the animals (Menz et al. 2023). In fact, the dose–response relationship of a genotoxic event, a mutation and any subsequent adverse outcome may be affected by various factors – such as tissue and cell specificity, individual variability in DNA repair capacity and developmental stage – which cannot be adequately addressed by standard testing methods. In general, this is true also for other endpoints. However, given the severity of potential effects, such as cancer formation, it is of particular relevance for genotoxic substances.

As a straightforward approach, and because of the classical association between genotoxicity and carcinogenicity, a number of studies investigated the association between PoDs derived from *in vivo* genotoxicity assays (generally micronucleus test and/or TGR assay) with those from carcinogenicity studies. Overall, a good correlation was observed, especially for tissue-matched PoDs. However, there was a pronounced scatter, spanning up to 1 or 2 orders of magnitude (Hernandez et al. 2011; Soeteman-Hernández et al. 2015). Nevertheless, from the available data it appears that genotoxicity-based PODs are in many cases lower than those of downstream endpoints, which introduces additional safety margins (MacGregor et al. 2015a; Chepelev et al. 2023). A thorough discussion of these aspects is available in the review by Menz et al. (2023).

In the early days, genotoxicity data were generally only considered as a predictor for (rodent) carcinogenicity. However, several decades ago, genotoxicity was recognised as a *bona fide* endpoint, as it may result in various adverse health effects (Health Canada 1993; Heflich et al. 2020; White et al. 2020). The induction of adverse effects by genotoxicants is of stochastic nature, *i.e.* the risk for an adverse event increases with increasing dose (Herber et al. 2001; Metruccio & Moretto 2018). Therefore, under the traditional paradigm, any exposure to a substance with proven genotoxicity was expected to increase the risk for human diseases and should thus be avoided, although the actual risk at human-relevant exposures is widely unknown and might indeed be negligible at low exposure levels. In the context of quantitative genotoxicity assessments, it is therefore desirable to more precisely describe the likelihood of health implications that are associated with a genotoxicity-based PoD, and their magnitude.

#### 2.1.3. Available methods

Standard genotoxicity tests come with a number of limitations in view of the quantitative interpretation of the dose–response data. Nevertheless, established and guideline conforming *in vivo* studies, such as the transgenic rodent somatic and germ cell gene mutation assays (OECD TG 488), the *in vivo* mammalian alkaline comet assay (OECD TG 489), the mammalian erythrocyte micronucleus test (OECD TG 474) or, the mammalian bone marrow chromosome aberration test (OECD TG 475), can provide reliable data for genotoxicity assessments, albeit to a different degree of toxicological relevance.

The MoA-based risk assessment approach requires data beyond the information obtained in standard (geno)toxicity studies. The generation of such data by guideline conforming animal tests is challenging, both in terms of technical complexity and animal welfare considerations. In this context, NAMs may prove to be a valuable alternative. As high-throughput/high-content approaches, they offer the potential to address several endpoints and to test numerous chemicals simultaneously across a wide dose range. In addition, currently developed *in vitro* NAMs are often based on human cell systems, increasing the toxicological relevance for humans. Regardless of the origin of a cell line, particular attention should be paid to aspects such as p53 status and metabolic competence (Kirkland et al. 2007). The current utilisation of *in vitro* NAMs for regulatory purposes is still in nascent stages (Felter et al. 2021), albeit some assays have reached contemporary levels of applicability and are currently assessed with regard to their regulatory use in the OECD working program. This includes ToxTracker (OECD Project 4.125), gH2AX (OECD Project 4.168), *in vitro* alkaline Comet assay (OECD Project 4.190), 3D reconstructed skin micronucleus test and Comet assay (OECD Project 4.139) and ecNGS (error-corrected next generation sequencing; also known as error-corrected sequencing, ECS) methods (OECD Project 4.175). Other, less developed NAMs could also make a significant contribution to a MoA-based risk assessment (*e.g.* transcriptomic biomarker such as GENOMARK and TGx-DDI) (Thienpont et al. 2024). In the field of biomedical research, a plethora of assays with high human relevance is available for characterising the MoA in a relatively straightforward manner (Stolz et al. 2020).

#### 2.1.4. Data interpretation

The use of AOPs to organise the available mechanistic information and to facilitate its use in chemical safety assessment was widely supported (Ankley et al. 2010). Although there is an ongoing effort to develop AOPs and AOP networks in the field of genotoxicity (Sasaki et al. 2020; Cho et al. 2022; Huliganga et al. 2022; Demuynck et al. 2025), the AOP Wiki so far only lists very few matured (WPHA/WNT endorsed) AOPs related to mutations as an adverse outcome or key event (KE) in the etiology of human diseases such as, *e.g.* cancer, reduced fertility, or genetic disorders in the offspring (information as per August 2025; https://aopwiki.org/). In the sequence of events that lead to an increase in mutations, inadequate DNA repair is a common key event (KE 155), which typically occurs when DNA repair systems are overwhelmed and repair of the substance-induced damages are no longer error-free. The AOP Wiki provides extensive information on various aspects of DNA repair, including examples on how to measure it. What is missing in the AOP framework, though, is knowledge of the quantitative relationship between the sequential KEs. How this knowledge, if available, could be utilised for a quantitative assessment has been illustrated by Fukushima and colleagues in their study on quantitative relationships between KEs in the process of tumour formation for the DNA-reactive carcinogens 2-amino-3,8-dimethylimidazo-(4,5-f)quinoxaline (MeIQx) and 2-amino-3,8-dimethylimidazo(4,5-f)quinoline (IQ) (Fukushima et al. 2018). By comparing PoDs (expressed as BMDL_10_) derived for KEs such as DNA adduct formation, mutation frequency, preneoplastic lesions and tumour formation, the authors noted that the BMDL_10_ values increase with each additional step towards cancer growth. The gap between the onset of DNA adduct formation and increased mutation frequency was particularly distinct, suggesting efficient repair capacities and a threshold below which DNA damages do not lead to an elevated mutation frequency. Although some of the methods applied are debatable (*e.g.* using the same CES/BMR across different endpoints), the study serves as a useful example of a quantitative assessment approach. Moreover, the results are consistent with data published by Soeteman-Hernández *et al.* (Soeteman-Hernández et al. 2015), indicating that earlier events are more sensitive than the corresponding downstream outcomes. Hence, sufficient mechanistic knowledge regarding the genotoxic MoA leading to DNA lesions and corresponding cellular capacities for their repair, ideally in combination with information on the dose required to trigger each sequential KEs, would be needed to substantiate a quantitative assessment. Following this rationale, a MoA-based risk assessment has been proposed for genotoxic carcinogens (Hartwig et al. 2020). As genotoxicity MoAs are diverse, this can only be done on a case-by-case basis for data-rich substances. Given the few known examples where a MoA-based quantitative assessment has been successfully applied, the approach may not be applicable to a large number of chemicals without massive improvements in not only high-throughput testing but also automation of data and network analysis.

A more pragmatic and less data-intensive approach can be followed if the genotoxic MoA for a substance is known, and there is general consensus that this particular genotoxic MoA has a threshold. A quantitative assessment for aneugens that interact with cellular components involved in cell division, is in general accepted by regulators. In 2021, EFSA published a guidance document to facilitate a quantitative approach specifically for aneugens (EFSA 2021b). However, other indirect, non-DNA-reactive mechanisms leading to genotoxic effects, such as, *e.g.* inhibition of DNA synthesis, interference with DNA repair or oxidative stress, are also of practical relevance in risk assessments (EFSA 2019b, EFSA 2024), and are in principle similarly subject to mechanistic thresholds, and should hence likewise be considered for quantitative assessments.

#### 2.1.5. Regulatory assessments that already integrated quantitative dose–response analysis from genotoxicity data

Although not implemented in routine regulatory assessments, there are a few examples of quantitative approaches that were either conducted or accepted by regulatory bodies.

The most prominent example in this regard is related to the accidental exposure of HIV patients to the known DNA-reactive genotoxic carcinogen ethyl methanesulfonate (EMS), caused by intake of EMS-contaminated antiretroviral medication. EMS directly reacts with the DNA, inducing alkylation of nucleotides at various sites. In line with the traditional approach for DNA-reactive genotoxicants, an appropriate assessment for EMS would be of qualitative nature. However, in 2007, HIV patients had been given Viracept (nelfinavir mesilate) that later became known as being contaminated with EMS, prompting a recall of the contaminated batches and a comprehensive investigation into the potential cancer risk related to EMS exposure (Müller & Singer 2009). The pharmaceutical company responsible for marketing Viracept (Hoffman-La Roche) conducted an assessment based on a large data set, far beyond standard regulatory requirements, *e.g.* including extensive *in vivo* experiments, and investigations into the genotoxicity MoA, and DNA repair mechanisms involved in protecting from EMS-induced mutations. Multiple lines of evidence supported the conclusion that a non-linear dose–response relationship can be assumed for the direct DNA-reactive alkylating agent EMS, which enabled a quantitative risk assessment and the derivation of a ‘safe level’ for EMS consumption – an approach that was eventually accepted by the European Medicines Agency (EMA) (Müller & Gocke 2009; Walker et al. 2009). Because several doses had been tested below the putative threshold for mutations, a No Observed Effect Level (NOEL) was derived from the dose–response curves obtained in a micronucleus and a lacZ-transgenic rodent assay in mice (MutaMouse). Moreover, multiple administrations of low doses did not give a response, whereas a single administration of a high dose – which corresponded to the total amount of all low-dose administrations – showed a clear mutagenic activity. Mechanistic reasoning explaining these observations was provided on the genotoxicity MoA and an understanding of cellular protective mechanisms, *e.g.* DNA repair. Based on an evident gap between those exposure levels leading to an increased formation of protein adducts – a biomarker for the internal exposure to the reactive species – and those that cause mutations, it was concluded that at low exposure levels EMS-mediated DNA damage can be repaired efficiently. Only when the repair capacities are exceeded, DNA adducts can no longer be efficiently eliminated and mutations occur (Gocke et al. 2009). Another line of evidence in support of efficient DNA repair and a threshold mechanism was later provided by *Pig-a* experiments in rats. Although the dose–response relationship at high concentrations was linear in the acute setting, additivity was not observed when the same total doses were fractionated and administered over 28 days (Dobo et al. 2011). Taken together, the assessment performed for EMS exemplified how to combine and integrate different streams of data, including robust *in vivo* mutagenicity data with increased statistical power together with information on the genotoxic MoA, DNA repair mechanisms and EMS exposure. On the basis of these data, the investigators derived an apparent practical threshold below which exposure to a DNA-damaging agent with a well-known MoA does not increase the mutant frequency (MF). From the quantitative interpretation of the data, it was concluded that for exposures with a sufficient margin from the derived PoDs the cancer risk is deemed negligible. Since exposure of patients taking the medication was well below the practical threshold, even under worst-case scenarios, no additional cancer risk was assumed (Müller & Gocke 2009; Müller et al. 2009). EMS was later tested in other *in vivo* models, such as *gpt-delta* transgenic mice and *Pig-a*, both of which are characterised by lower MFs in their respective vehicle controls, yielding PoDs up to five times lower than the initially derived PoDs from the *lacZ* model (Cao et al. 2014). Hence, it was concluded that models with low spontaneous background MF may be preferred (MacGregor et al. 2015a). However, the deviating results also underline the inherent uncertainties that arise from quantitative interpretations of genotoxicity data. Furthermore, there was consensus that the conclusions drawn for EMS may not necessarily hold true for other alkylating agents. For example, at least under the study conditions used, no threshold could be derived from the data obtained with ethylnitrosourea (ENU), which was tested in the same study (Gocke et al. 2009). Extrapolating and generalising the EMS results to genotoxicants with MoAs other than alkylation may even be less appropriate. Nevertheless, the EMS case demonstrated that adequate data and interpretation procedures are fundamental requirements to diverge from the default risk assessment approach (Lutz 2009).

The Committee for Risk Assessment (RAC) of the European Chemicals Agency (ECHA) used quantitative mutagenicity data to derive an Occupational Exposure Limit (OEL) for the genotoxic carcinogen benzene (ECHA 2018). Benzene causes structural and numerical chromosomal aberrations which are believed to play a major role in the etiology of leukaemia in exposed workers. Based on the WoE related to genotoxic modes of action, RAC considered benzene-induced chromosomal damages to be predominately a result of secondary indirect mechanisms characterised by a non-linear dose–response relationship. Consequently, RAC concluded that an OEL based on chromosomal damages is protective and without a significant residual cancer risk. The conclusion was later supported by a case study, employing a next generation framework to quantitatively assess human health risk based on genotoxicity data (Luijten et al. 2020).

Even in situations where comprehensive data are available, some uncertainties will always remain. However, the regulatory key question is whether the available data are sufficient to allow for a toxicological conclusion that should be supplemented with a description of the inherent uncertainties. A data-driven MoA-based approach may be sufficient to reduce uncertainty to a level acceptable for risk management.

There are other examples in the literature that have not been scrutinised with respect to their regulatory acceptability and thus, may or may not be adequate for quantitative considerations in a regulatory context. However, they demonstrate how quantitative interpretation of genotoxicity data may be achieved based on mechanistic information. For example, in the chain of events leading to mutations and more apical adverse health effects, each KE may be triggered at different doses, with or without a threshold. Efficient repair capacities are considered to be a reason for a threshold below which DNA damage does not lead to an increased mutation frequency (Fukushima et al. 2018). Evidence for sufficient DNA repair has also been utilised to justify the derivation of regulatory human exposure limits such as PDE values based on PoDs from *in vivo* genotoxicity dose–response curves that have been derived for alkylating nitrosamines, such as *N*-nitrosodimethylamine (NDMA), *N*-nitrosodiethylamine (NDEA) (Johnson et al. 2021), and *N*-nitroso-bisoprolol (Simon et al. 2025). These substances are direct DNA-reactive genotoxicants that are able to form DNA adducts. Other examples of MoA-based assessment of genotoxic compounds, such as acrylamide, aflatoxin B1 or arsenic, are thoroughly compiled by Hartwig *et al.* alongside a proposal for an assessment strategy for genotoxic carcinogens (Hartwig et al. 2020).

From examples as discussed above, it became apparent and was unanimously agreed to, that information on the genotoxic MoA including mechanistic understanding about formation (direct or indirect mechanism) and repair (*e.g.* saturation of DNA repair capacities) of chemical-induced DNA damages is of utmost importance for quantitative interpretations. To this end, the WoE should be thoroughly evaluated as for many mutagens, multiple genotoxic MoA have been described.

In the workshop it was discussed that existing tests and novel NAMs may be combined in a synergistic manner, with the objective of making a significant step forward in the field of genotoxicity risk assessment and transitioning from a hazard identification to a hazard characterisation approach. Although the standard test battery primarily serves for hazard identification, it would be followed by a detailed characterisation of the substance in a second tier using a combination of NAMs addressing different KEs. The resulting data could be evaluated in a WoE approach, based also on previous knowledge captured in AOP networks, to determine the feasibility of a quantitative assessment and the necessity of follow-up *in vivo* studies. Guidance on WoE assessment considering this additional information would be needed in order to help registrants and assessors to take more informed decisions.

### 2.2. What is needed to build confidence in quantitative approaches?

An important objective of the BfR symposium was to identify the requirements that would enable or improve the acceptance of quantitative assessments of genotoxic compounds by regulatory authorities and conceptualise a roadmap. As the quantitative aspect has not played a major role in the assessment of genotoxicity in the past, the currently established assessment strategy was reviewed and a number of scientific and regulatory aspects were discussed that should be considered when performing quantitative assessments. Overall and as outlined above, quantitative assessments have so far only been accepted in specific cases and thus, performing quantitative risk assessments is challenging at the moment. However, there is also a consensus that quantitative and/or semi-quantitative risk assessments in genotoxicity is the way forward and that there is an opportunity and urgent need in situations where exposure to genotoxic substances cannot be avoided (*e.g.* genotoxic contaminants in food). To advance quantitative approaches and to gain acceptance by regulatory authorities, a number of aspects were identified that need to be addressed to enable quantitative assessment.

Considerable uncertainties arise from the use of currently established standard genotoxicity studies, both for hazard identification but even more for quantitative interpretations. These are in many cases restricted to certain tissues/organs, for example the *in vivo* micronucleus test according to OECD TG 474 needs exposure of the bone marrow. The PoD of a genotoxic effect, however, may considerably vary between different tissues, *e.g.* depending on toxicokinetic and other tissue-specific factors. In addition, standard genotoxicity tests can only cover specific genotoxic MoAs. Furthermore, treatment duration and sampling time are determinants influencing the quantitative outcome. In turn, PoDs that are derived from different genotoxicity studies can vary considerably, *e.g.* depending on the genotoxicity endpoint, target tissues/organs, variability, and study conditions. Results can span more than one order of magnitude. Of note, the dose–response relationship of a genotoxic event, a mutation and any subsequent adverse outcome may be affected by various factors – such as tissue and cell specificity, individual variability in DNA repair capacity and developmental stage – which cannot be adequately addressed by standard testing methods (Long et al. 2016; Guérard et al. 2017; Wills et al. 2017; White et al. 2020; Marchetti et al. 2021; Mittelstaedt et al. 2021; Menz et al. 2023). In this regard, clear and internationally harmonised guidance on how to address these uncertainties when using genotoxicity-based PoDs is urgently needed. In addition, novel techniques like ecNGS may help to overcome at least some of these uncertainties in future. There is also a need for a more targeted approach in future assessment. For example, a standard *in vitro* battery may, like today, be used for hazard identification followed up by NAM-based evaluation addressing various aspects (human relevance, MoA, kinetics, target tissue, repair capacity) and – if necessary – higher tier follow up testing (*e.g.* 3D *in vitro* models if available/suitable, *in vivo* assays). On that note, a stepwise IATA (integrated approach to testing and assessment, *i.e.* combining multiple sources of information to conclude on the toxicity of chemicals) has already been proposed by Health Canada (Felter et al. 2021).

It is generally acknowledged that BMD modelling is an appropriate way to derive PoDs for genotoxic effects and should also be taken into account in the test guideline development. To effectively apply BMD modelling, certain test design modifications would be helpful, such as increasing the number of dose groups and carefully selecting the dose range. A tiered approach was recommended to capture low-dose effects accurately. Since BMD modelling is a critical step when deriving a BMD as a PoD (WHO 2020a; EFSA 2022), harmonisation of this procedure for genotoxic endpoints was considered helpful to ensure consistency and reliability.

Another critical aspect is the determination of appropriate benchmark response (BMR) values for various genotoxicity endpoints. An additional workshop with relevant stakeholders, including ECHA, EFSA, and EMA, to establish consensus BMR values for genotoxicity should be organised. Ongoing case studies on BMRs for different genotoxic endpoints and tests provide the basis for these discussions (White et al. 2025).

Additionally, a comprehensive and systematic analysis of toxicological PoDs derived from genotoxicity and carcinogenicity data was considered beneficial for gaining deeper insights into the mechanisms, dose-dependency of effects, and potential thresholds of genotoxic substances. Such comparisons could also help better characterise cancer risk associated with genotoxic effects. A dedicated working group within the HESI Genetic Toxicology Technical Committee (GTTC) is addressing this aspect.

Finally, as a very important issue, the lack of standardised guidance to support quantitative approaches in genotoxicity assessments was highlighted. It was emphasised that harmonised guidance documents and testing protocols need to be developed that align with current scientific standards and are based on validated research. Adapting the existing OECD Test Guidelines, which are currently designed for qualitative assessments and therefore are less suited for quantitative analysis, was also discussed.

## 3. Way forward

Fig. 2 presents an overview on the status quo as well as on short-term and long-term milestones for a roadmap towards the quantitative interpretation of genotoxicity data in the risk assessment of chemicals.

## 4. Overall learnings from the symposium and conclusions

Taken together, many aspects of a quantitative interpretation of dose–response data from genotoxicity studies were discussed during the BfR symposium. The following section summarises key issues to consider for successful implementation of quantitative genotoxicity assessment strategies without negatively affecting human health protection. These aspects have been widely shared by the workshop participants and also reflect the discussion in the regulatory community.

- There is still consensus to consider genotoxicity as an independent *bona fide* endpoint. An increasing demand for a paradigm shift towards a more quantitative assessment of genotoxic substances, away from the current binary classification, is acknowledged by the majority of scientists in the field of genotoxicity.
- In certain regulatory contexts, a quantitative assessment of genotoxicity data will be beneficial over the traditional approach as it provides potency information. It thereby could enable a more relevant risk assessment, and supports risk managers in making decisions.
- There is consensus on technical aspects of evaluating dose–response data from genotoxicity studies. Disagreements and controversies mainly concern the toxicological relevance of PoDs derived from such data and related methodological detail. Therefore, collective efforts of all relevant stakeholders are needed to improve genotoxicity assessment without compromising on the protection of human health. This could be addressed in dedicated follow-up activities involving all relevant stakeholders.
- Two different approaches to quantitative interpretation of genotoxicity data have been conceptualised. The feasibility of either approach strongly depends on the regulatory context and the availability of appropriate data. A MoA-based interpretation may be considered appropriate for data-rich compounds for which the available data demonstrate a negligible effect at low doses. Also, what constitutes a negligible effect that may be accepted needs to be defined and harmonised in this context. A quantitative assessment may be helpful for substances where exposure cannot be avoided and only *in vivo* genotoxicity data are available. In such a scenario, PoDs could be derived in a pragmatic approach on the basis of data from already established standard *in vivo* tests (*e.g.* micronucleus test, transgenic rodent assay, *Pig-a* assay, Comet assay). It might be appropriate to use these PoDs for MoE calculations to get an idea of the concern that is associated with a given exposure. However, for intentionally used substances where exposure is avoidable, the pragmatic approach or even the establishment of HBGVs was not generally supported by the participants of the symposium.
- The dose–response relationship of direct and indirect acting genotoxicants is likely to be non-linear, not the least because of the biology of the underlying mechanisms. Thus, a threshold below which the response does not exceed any background level may be established. Mechanistic understanding is essential in this context: regardless of whether a substance is considered as a direct or indirect genotoxicants, its MoA should be clearly characterised to inform risk assessment to justify the threshold approach. This ideally also includes information on the toxicokinetics of the substances, indicating the potential for systemic exposure and exposure of potential target organs/tissues/cells.
- Structuring mechanistic information within the AOP framework could standardise the assessment, unveil data gaps, and ultimately foster regulatory acceptance. Expanding AOPs for genetic toxicity with quantitative information related to the relationship between key events and linking these into AOP networks provides an alternative concept for quantitative assessment. NAMs addressing established KEs, thus yielding information on the MoA would significantly improve the assessment for both qualitative and quantitative evaluations.
- Although additional *in vivo* assays with more doses and animals would certainly reduce the uncertainty in the assessment, there are reasons opposing a call for more extensive animal testing, including ethical reasons, time and resource constraints, or human relevance.
  This results in strong demand to include *in vitro* data and human (epidemiological) data in the risk assessment process. Modelling approaches, *e.g.* PBTK/IVIVE, may enhance the relevance of assessments in the future. Furthermore, more targeted *in vitro/in silico* initial testing strategy may be developed to categorise genotoxicants according to *e.g.* potency, MoA (direct/indirect mechanism, DNA repair capacity, genotoxic endpoint), or toxicokinetic properties. This may help in choosing an appropriate follow-up strategy including quantitative considerations.
- A quantitative assessment of genotoxicity data is considered appropriate for compounds where the available data demonstrates a thresholded MoA. For aneugens such quantitative approaches have already been addressed in regulatory guidance documents.
- There is still a need for further research, *e.g.* on the quantitative association between genotoxic effects and apical endpoints like carcinogenicity, options for integrating *in vitro* data and NAM data, appropriate uncertainty/assessment factors, and the usefulness of novel techniques like ecNGS.
- Quantitative risk assessment is particularly beneficial where exposure to genotoxic substances cannot be avoided without unreasonable effort, or if associated with the loss of a significant societal benefit (*e.g.* unavoidable genotoxicants in foods). Therefore, in sectors regulating unavoidable substances like contaminants, the transition towards quantitative approaches may be more easily achievable as compared to other regulatory areas.
- The uncertainties associated with quantitative assessment need to be thoroughly evaluated. Also, vulnerable groups of the population should be taken into consideration in this context. The introduction of quantitative approaches into genotoxicity assessment must not be at the expense of human health protection. In addition, a quantitative assessment may lead to a situation in which a residual, albeit small risk, needs to be appropriately communicated and accepted. However, the level of acceptable risk is rather a societal and/or political than a toxicological aspect and should not be neglected in the discussion. This is particularly challenging because the prevailing public and political discussion on this issue has often hindered objective scientific debate. Yet, policy makers already have to make informed decisions on the potential risks from genotoxicants and mutagens and are likely to do so more often in the future. To some extent, this is already reflected in EU regulation, for example with the generic concentration limits for substances triggering classification of mixtures or multiconstituent substances (*i.e.* substances with impurities or additives).
- From a regulatory perspective, the development of guidance documents outlining the process for quantitative assessments is essential to ensure harmonisation of procedures and increase regulatory acceptance.

## Figures and Tables

**Fig. 1.**
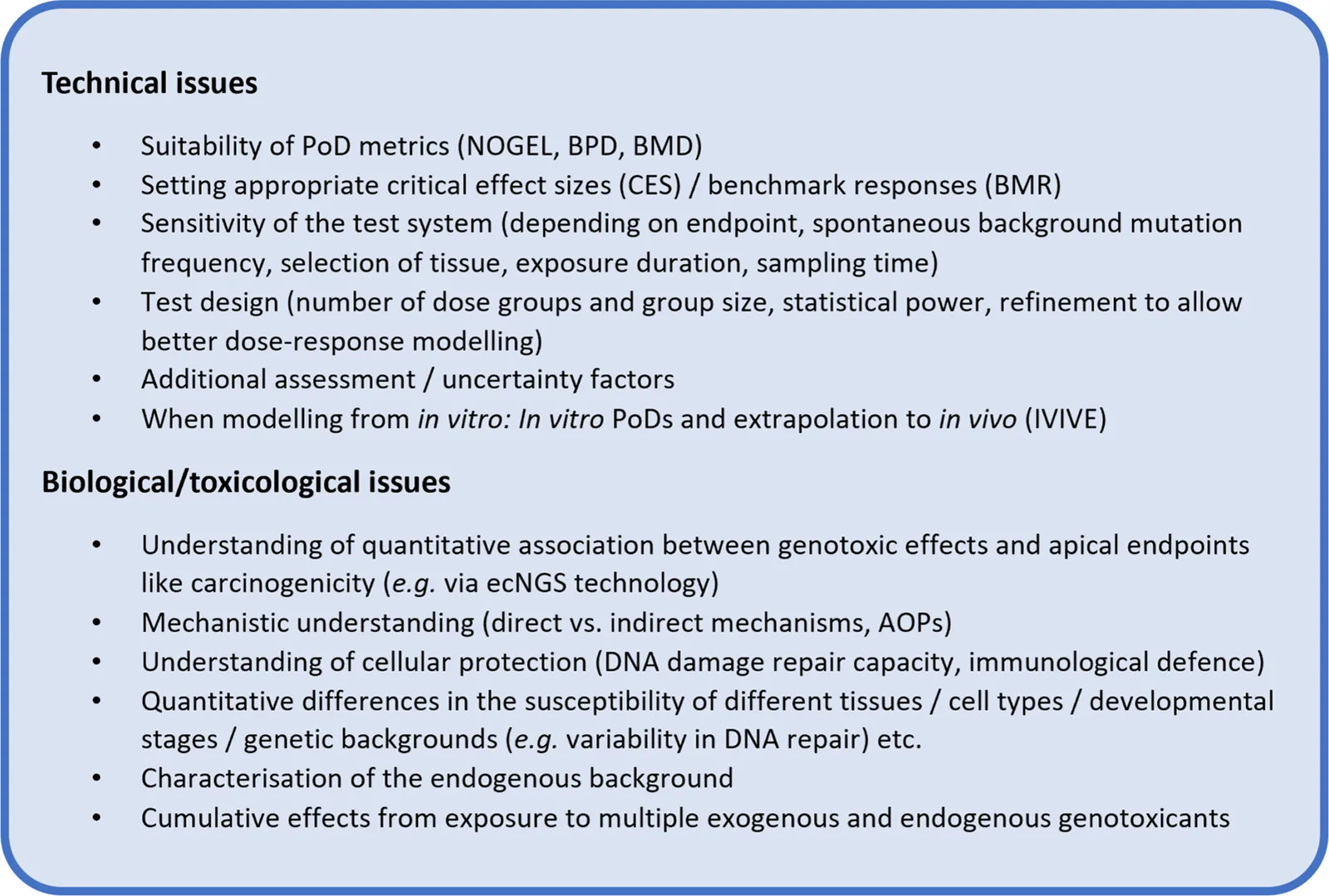
Summary of technical and biological/toxicological issues relevant in the discourse Abbreviations: PoD, point of departure; NOGEL, no observed genotoxicity effect level; BPD, breakpoint dose; BMD, benchmark dose; IVIVE, *in vitro* to *in vivo* extrapolation; ecNGS, error-corrected next generation sequencing; AOP, adverse outcome pathway.

**Fig. 2.**
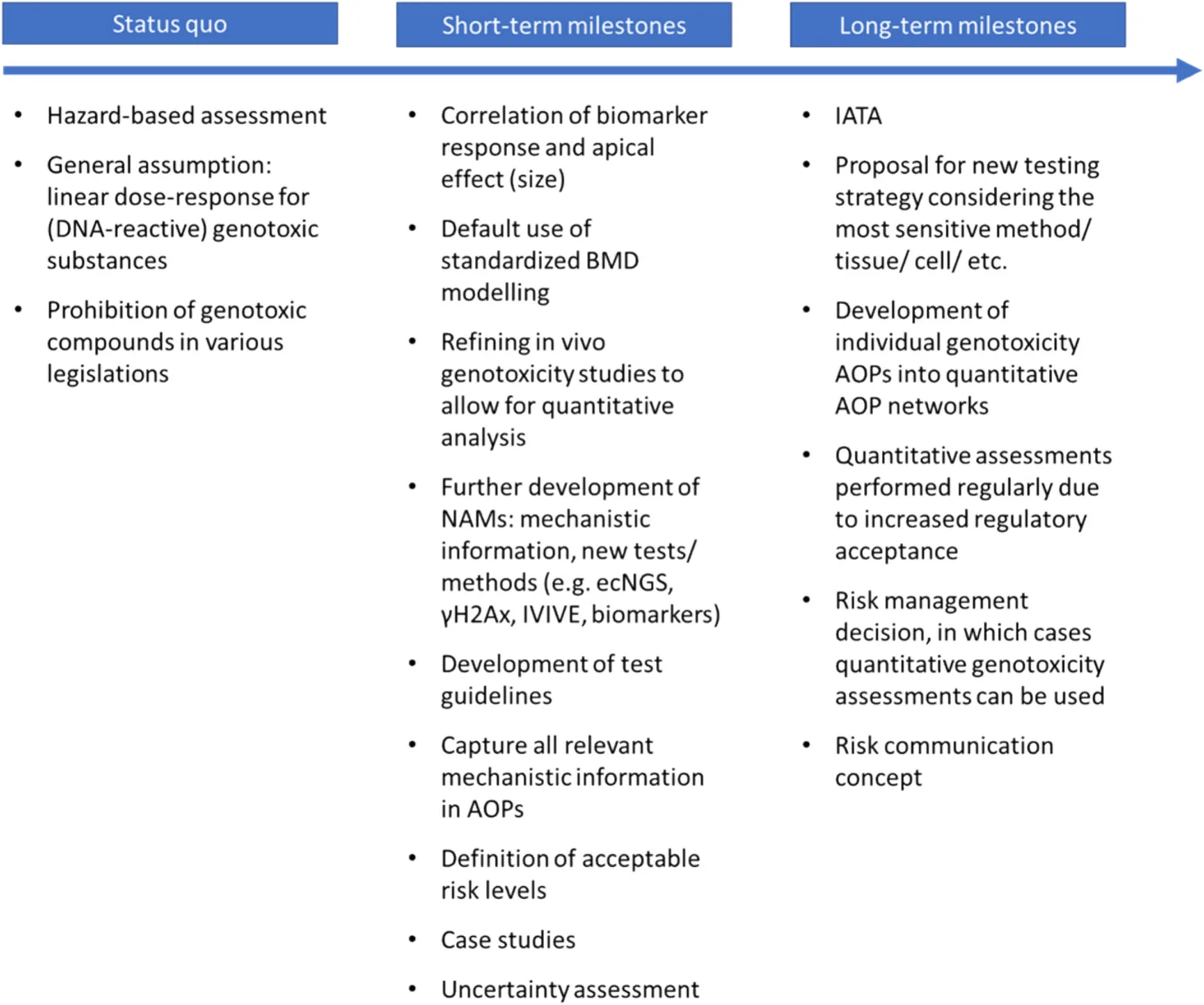
Status quo and milestones for a roadmap towards the quantitative interpretation of genotoxicity data.

**Table 1:** Overview on aspects related to hazard and risk assessment procedures for genotoxic compounds for selected regulatory areas in the EU.

Regulatory areas	Regulation	Hazard assessment of genotoxic substances	Risk assessment of genotoxic substances	Selected regulatory consequences

Substances as such or in mixtures	Regulation (EC) No 1272/2008	According to classification criteria in CLP regulation (Muta 1A, 1B or 2)	Not applicable	No classification/ labelling of mixtures as Muta if mutagenic ingredient is below GCL^a^ (**Muta 1A/B**: 0.1 % w/w; **Muta 2** 1 %) No guidance available for derivation of SCL^b^
Substances as such, in mixtures or articles	Regulation (EC) No 1907/2006	According to classification criteria in CLP regulation (Muta 1A, 1B or 2)	Calculation of DNEL based on PoD if threshold-based MoA can be clearly demonstrated Calculation of DMEL based on cancer data if genotoxicity is relevant mechanism for cancer development (cancer risk of 1 per 1.000.000 individuals) Else: qualitative consideration for avoiding exposure to the substance	**Muta 1A,1B**: shall not be placed on market as such or mixtures for supply to public above SCL^b^ or GCL^a^ (Annex XVII restriction, entry 29) **Muta 1A, 1B:** easy listing as SVHC^c^ (authorisation process could be linked)
Substances in plant protection products	Products Regulation (EC) No 1107/2009	According to classification criteria in CLP regulation (Muta 1A, 1B or 2)	Threshold accepted for aneugens. Concern over mutagenicity is sufficient to abstain from derivation of reference values	**Muta 1A, 1B:** Non-approval (no derogation) **Muta 2:** Approval only if threshold-based MoA can be clearly demonstrated, as only then reference values are applicable for risk assessment
Substances in biocidal products	Regulation (EU) 528/2012	According to classification criteria in CLP regulation (Muta 1A, 1B or 2)	Reference value or MoE approach based on PoD if threshold-based MoA can be clearly demonstrated Calculation of DMEL or application of large assessment factor approach in cases of derogation Concern over mutagenicity is sufficient to abstain from derivation of reference values	**Muta 1A, 1B:** Non-approval (possible derogation under specific conditions such as negligible risk, absence of alternative or disproportionate societal impact) **Muta 2:** No authorisation of products for use by general public
Cosmetic ingredients	Regulation (EC) No 1223/2009	According to the SCCS Notes of Guidance in its most actual version (SCCS 2023)		**Muta 1A,1B & 2:** The Regulation prohibits the use of such substances in cosmetic products, unless defined criteria are cumulatively met including their use is evaluated and found safe by the SCCS^d^
Food contact materials	Regulation (EC) Nr. 1935/2004		Materials and articles must not transfer under normal or foreseeable conditions of use their constituents to food in quantities, which endanger human health.	A migrating substance (transfer from packaging into food) should not be genotoxic. Genotoxic substances can be used in food contact materials, if no migration occurs or a risk assessment found the use safe for consumers.
Food additives	Regulation (EC) No 1333/2008		Food additives can only be authorised if “it does not, on the basis of the scientific evidence available, pose a safety concern to the health of the consumer at the level of use proposed”.	If there is a concern for genotoxicity on the basis of *in vivo* data, food additives cannot be authorised.
Feed additives	Regulation (EC) No 1831/2003		“Feed additives shall not have an adverse effect on animal health, human health or the environment”	If there is a concern for genotoxicity on the basis of *in vivo* data regarding the target animals or the consumers of food of animal origin, feed additives cannot be authorised.
Flavourings	Regulation (EC) No 1334/2008		Flavourings can only be authorised if “they do not, on the basis of the scientific evidence available, pose a safety risk to the health of the consumer”.	If there is a concern for genotoxicity on the basis of *in vivo* data, flavourings cannot be authorised.
Food contaminants	Council Regulation (EEC) No 315/93 Regulation (EU) No 2023/915		“Food containing a contaminant in an amount which is unacceptable from the public health viewpoint and in particular at a toxicological level shall not be placed on the market.” “Furthermore, contaminant levels shall be kept as low as can reasonably be achieved by following good practices at all the stages [...].” “The food listed in Annex I shall not be placed on the market and shall not be used as a raw material in food or as an ingredient in food where it contains a contaminant at a level which exceeds the maximum level set out in Annex I.”	ALARA: Risk mitigation measures as appropriate, *e.g.* maximum levels that are exposure driven
Pharmaceuticals	Directive 2001/83/EC	Determination whether compound is non-genotoxic or genotoxic	Benefit/risk assessment for genotoxic compounds	Deny of approval in case of negative benefit/risk evaluation

Verbatim quotes in this table are taken from the respective regulation indicated in the same row.

a GCL- generic concentration limit.

b SCL- specific concentration limit.

c SVHC- substances of very high concern.

d SCCS- scientific committee on consumer safety.

## Data Availability

No data was used for the research described in the article.
